# The 3D Reconstruction of *Pocillopora* Colony Sheds Light on the Growth Pattern of This Reef-Building Coral

**DOI:** 10.1016/j.isci.2020.101069

**Published:** 2020-04-18

**Authors:** Yixin Li, Tingyu Han, Kun Bi, Kun Liang, Junyuan Chen, Jing Lu, Chunpeng He, Zuhong Lu

**Affiliations:** 1 State Key Laboratory of Bioelectronics, School of Biological Science and Medical Engineering, Southeast University, Nanjing 210096, China; 2 Nanjing Institute of Paleontology and Geology, 39 East Beijing Road, Nanjing 210008, China; 3 Key Laboratory of Vertebrate Evolution and Human Origins of Chinese Academy of Sciences, Institute of Vertebrate Paleontology and Paleoanthropology, Chinese Academy of Sciences, PO Box 643, Beijing 100044, China; 4 CAS Center for Excellence in Life and Paleoenvironment, Beijing 100044, China

**Keywords:** Marine Organism, Biological Sciences, Methodology in Biological Sciences

## Abstract

Coral reefs are formed by living polyps, and understanding the dynamic processes behind the reefs is crucial for marine ecosystem restoration. However, these processes are still unclear because the growth and budding patterns of living polyps are poorly known. Here, we investigate the growth pattern of a widely distributed reef-building coral *Pocillopora damicornis* from Xisha Islands using high-resolution computed tomography. We examine the corallites in a single corallum of the species in detail, to interpret the budding, growth, and distribution pattern of the polyps, to reconstruct the growth pattern of this important reef-building species. Our results reveal a three-stage growth pattern of *P. damicornis*, based on different growth bundles that are secreted by polyps along the dichotomous growth axes of the corallites. Our work on the three-dimensional reconstruction of calice and inter-septal space structure of *P. damicornis* sheds lights on its reef-building processes by reconstructing the budding patterns.

## Highlights

• We use high-resolution computed tomography to investigate coral forming and polyp budding processes
• The calice reconstruction shows coral growth patterns and budding information
• Our work visualizes the growth pattern of *Pocillopora damicornis*
• High-resolution computed tomography is a method for future reef-building coral studies

## Graphical Abstract

**Figure:**
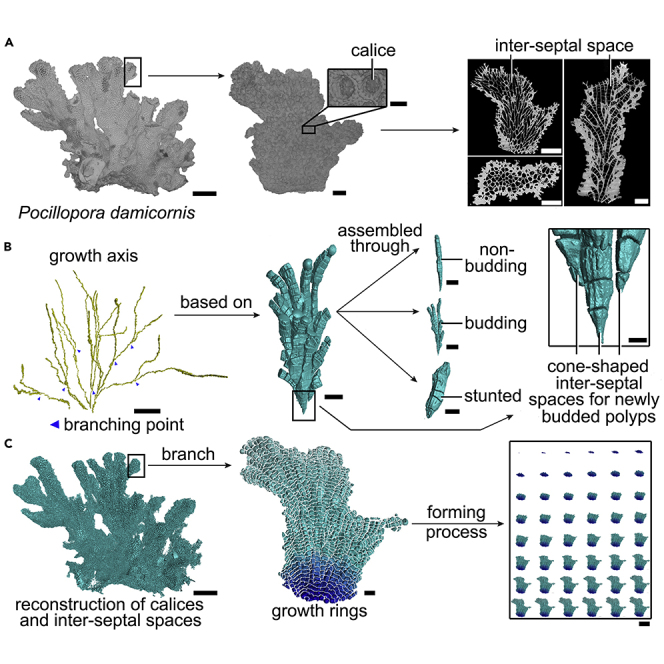

## Introduction

Reef-building scleractinian corals provide complex three-dimensional niches for various species (Costanza et al., 2014, Ellison et al., 2005, Knowlton et al., 2010, Weis et al., 2008, Graham, 2014). However, coral reefs are currently declining globally because of the climate change (Carpenter et al., 2008, Kleypas et al., 1999, Maynard et al., 2015), especially owing to the intensification of El Niño (Jokiel and Coles, 1977, Bellwood et al., 2004, De’ath et al., 2012), water-quality deterioration (Tambutté et al., 2015, Mollica, 2018), and overexploitation (Natt et al., 2017, Robinson et al., 2017). Studies on the biochemical processes governing coral reef and their ecological health under environmental changes are therefore undoubtedly in the spotlight (Fabricius et al., 2010).

The reef-building scleractinian coral *Pocillopora damicornis* is widespread in the Indo-Pacific ocean and is one of the most abundant and widely distributed species in the world (Veron and Staffordsmith, 2000). This coral has a high growth rate, which is advantageous in the competition to survive (Cunning et al., 2018). *P. damicornis* has stronger defenses against bleaching and is better adapted to environmental changes than most other coral species (Carpenter et al., 2008). In some regions of Xisha Islands where external disturbances such as the El Niño and ocean pollution have overwhelmed the capacity of corals to recover from damage, some stress-tolerant corals like *P. damicornis* show better environmental adaptability and become the dominant coral species instead of the most common species like *Montipora* and *Acropora* belonging to the family *Acroporidae* (Kayal et al., 2015, Bramanti and Edmunds, 2016, Adjeroud et al., 2018). This makes *P. damicornis* an important animal, and various aspects concerning this species should be studied, such as their speciation (Johnston et al., 2017, Schmidt-Roach et al., 2014), reproduction (Miller and Ayre, 2004, Schmidt-Roach et al., 2012, Combosch and Vollmer, 2013), symbiosis (Cunning and Baker, 2012, Kopp, 2015, Brener-Raffalli et al., 2018), and population genetics (Stoddart, 1984, Souter et al., 2009, Thomas et al., 2017). Recently, researchers have performed comprehensive studies on *P. damicornis* including their genomics (Torda et al., 2013, Combosch and Vollmer, 2015, Wilkinson et al., 2015, Mass et al., 2016, Crowder et al., 2017, Yuan et al., 2017, Zhang et al., 2018), polyp metabolism (Lecointe et al., 2013, Kvitt et al., 2015), zooxanthellae (Zhou et al., 2017), disease resistance (Ben-Haim et al., 2003), adaptation to environmental change (Crowder et al., 2014, Rivest and Hofmann, 2014, Paz-García et al., 2015, Rodríguez-Villalobos et al., 2016, Traylor-Knowles, 2016, Zhou et al., 2016, Zhou et al., 2018, Yu et al., 2017), and integration with microfluidic systems (Helman et al., 2008, Mass et al., 2012, Shapiro et al., 2016) and electronic sensors (Szabó et al., 2014, Mu et al., 2017).

However, although *P. damicornis* is one of the most extensively studied coral species in terms of overall macromorphological and microarchitectural details (Schmidt-Roach et al., 2013, Veron and Pichon, 1976), the structures of calice and inter-septal space and the relationship between coral growth pattern and its skeleton are still poorly known owing to the limitations of the technology. Most studies on coral structures are based on small samples at low resolutions. These studies are limited by the equipment such as optical microscopes (Welsh et al., 2017), scanning electron microscopes (SEMs) (Bonesso et al., 2017), and industrial computed tomography with low resolution (Chindapol et al., 2013), which did not provide a precise and comprehensive overview on the coral structures owing to limited resolution and scales. To study the growth patterns of the corals, traditional micro-CT has been used in several studies (Beuck et al., 2007, Knackstedt et al., 2006, Kruszynski et al., 2006, Kruszynski et al., 2007, Nishikawa et al., 2009). However, as micro-CT technology was still developing when these studies were performed, the reconstructed images revealed only the exterior of the coralla instead of detailed internal calice structures and growth patterns.

Recently, with the development of modern technology, high-resolution computed tomography (HRCT) has gained increasing attention in biological research for its high-resolution and nondestructive nature. Previous works based on HRCT on the internal skeletal structures of certain corals increase our knowledge on the coral skeletons (Janiszewska et al., 2011, Janiszewska et al., 2013, Tambutté et al., 2015). However, owing to lack of virtual segmentation and further investigation, our understanding of the 3D skeletal structures of the corals remains poor. Here, we investigate the three-dimensional skeletal structures of reef-building coral *P. damicornis* from Xisha Island, China, and reconstruct its calice and internal inter-septal space network by using HRCT and virtual segmentation, which helps us to understand the budding, growth, and distribution information of the polyps during the growth process (Figure S1).

## Results

### General Morphological Structure of *P. damicornis*

**Figure 1:**
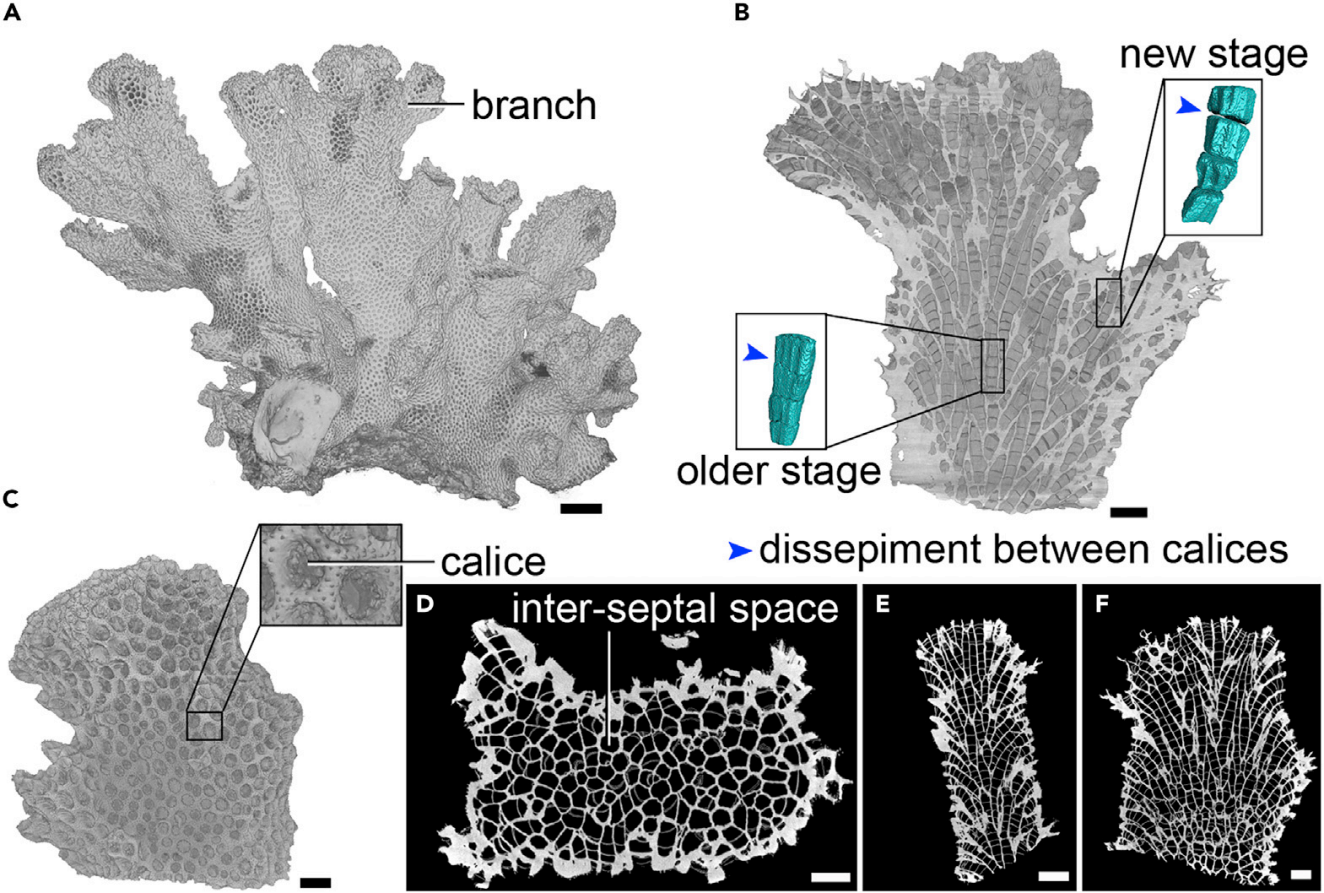
High-Resolution Micro-CT Reconstruction of *P. damicornis* (A) Reconstruction skeleton image of the entire *P. damicornis* sample. (B) Front vertical sectional image of branchlet one, with two calice and inter-septal space reconstructions. We reconstructed and compared two structures, including a newly built one at the edge and an older one deep inside the colony. There are apparent separations between the newly formed calices and inter-septal spaces, whereas the separations are not distinct between older inter-septal spaces. (C) 3D reconstruction of branchlet two and the calice of its surface polyp. By measuring the diameter at the opening of the hole, it is possible to directly obtain visual measurement data such as the diameter of polyp oral surface in a natural, undistorted state. Diameter of polyp abactinal surface and vertical height can be seen in Figure S3. This study effectively determines the volume of polyp calice and body size, which cannot be determined by polyp bailout or SEM (see Figure S3 for full image). (D) Top view image of the *P. damicornis* branchlet reconstruction model. (E) Lateral sectional image of the *P. damicornis* branchlet reconstruction model. (F) Front sectional image of the *P. damicornis* branchlet reconstruction model. Scale bars: (A) 1 cm; (B) 2 mm; (C) 2 mm; (D–F) 2 mm.

We assembled a 3D morphological structure incorporating details at both macroscopic and microscopic scales by reconstructing a single *P. damicornis* corallum with a size of approximately 3,600 cm^3^ (Figure 1A). The reconstructed image presents the complete form of the original coral skeleton details of surface bulges and calices, which significantly facilitates the study of its skeleton and biological characteristics. There are multiple calices closely packed on the surface, and a single polyp grows in each calice. The connections between the calices are realized by desmocytes that fasten the coral soft tissue to the skeleton. Coralla of *P. damicornis* have many branches, and the gaps between these branches are often the habitat of small aquatic organisms such as shrimps and crabs (Figure 1A).

To study the internal calices more clearly, we scan two small branches from a large corallum of *P. damicornis* and reconstruct their 3D structures at a high resolution (Figures 1B and 1C) to further study the calices and internal inter-septal spaces from the microscopic perspective.

### Skeletal and Calice and Internal Inter-Septal Space Structures of *P. damicornis*

To observe the skeleton and internal calices, we obtained 3D, cross-sectional, and slice images at multiple branches of the reconstructed *P. damicornis* models (Figures 1C–1F and S2A). There are no holes among the coral coenosteums, and each polyp has its own distinguishable calice, which is completely sealed off. Thus, there is no direct connection between adjacent calices. All these calices and inter-septal spaces record the spatiotemporal growth of each individual polyp in this colony. Owing to separation of tabulae, the living polyps are present only at the surface of the colony, and the interior spaces do not contain living polyps. Additionally, the corallites are much thicker than the coenosteums among all these skeleton structures (Figures 1D–1F).

By measuring the diameter on the top of the calice, we calculated the corallite diameter (Figure 1C). We also obtain the aboral surface diameter and speculate the height of polyps using these measurements (Figures S3A–S3C), therefore, to determine the calicular volume between last tabula and the calicular margin, which cannot be determined by polyp bailout or SEM techniques (Figures S3D–S3F). According to the reconstructed model, the diameters of both the oral and aboral surfaces are generally between 0.5 and 1 mm, and the polyp heights are between 0.1 and 0.6 mm.

In the reconstructed 3D images, bamboo-like calice and inter-septal space structures on the same branch of *P. damicornis* branch out in a generally alternate way (Figure S2A). We selected one part of the coral skeleton to reconstruct a 3D model. To show the internal structure of the coral, we dissected the selected coral skeleton along its vertical axis, revealing the internal bamboo-like structures; for more detailed observations, we also investigated a vertical section (Figure S2B).

### Growth Patterns and Internal Network of *P. damicornis*

**Figure 2:**
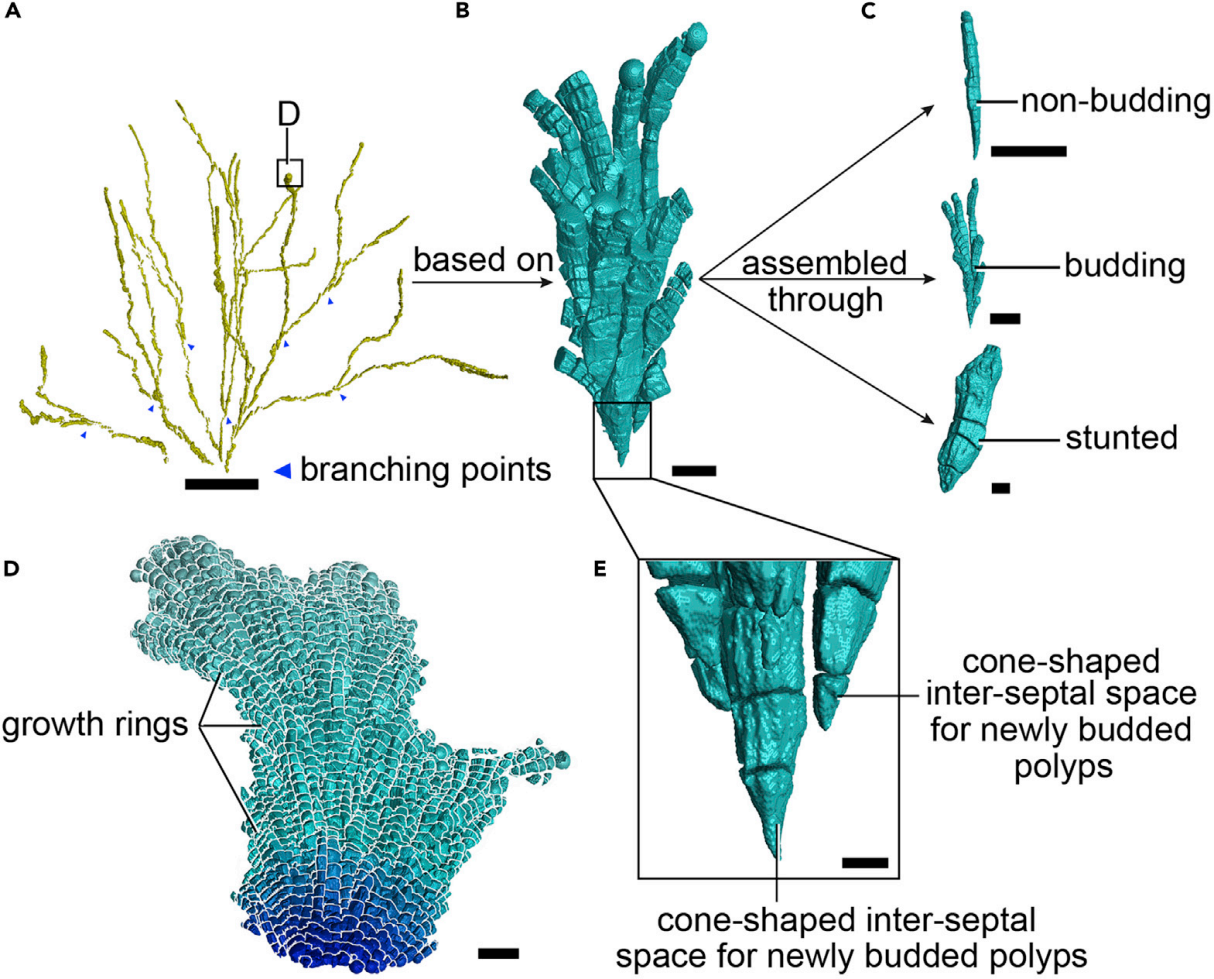
Growth Pattern of *P. damicornis* (A) The entire calice and inter-septal space that grows from a growing point to the surface of the coral of the *P. damicornis* branchlet showing the three-stage growth pattern of *P. damicornis* polyps. At this scale, the branching of *P. damicornis* is dichotomous, growing generally in two directions with many smaller branches budding from minor growth axes. The blue arrows mark the branching points of the growth axes. (B and C) (B) The growth bundle, which is the core structure of coral growth, based on growth bundles along the dichotomous growth axes, assembled through multiple growth types (C). (D) Growth rings of *P. damicornis*. (E) Amplifying lower edge of the *P. damicornis* growth bundle, showing the newly divided calices growing into larger column-shaped structures as polyp growth volume increases, the key feature for distinguishing the budding branch and spatial distribution of coral polyps, and is the basis for constructing the *P. damicornis* calice and inter-septal space network in virtual 3D reconstructions. Scale bars: (A) 1 cm; (B) 1 mm; (C) 500 μm; (D) 2 mm; (E) 200 μm.

To investigate the growth pattern of polyps, we reconstructed the calices and inter-septal spaces chamber by chamber (Figures 2, S4, and S5). The reconstructed results suggest that the coral skeleton can be divided into two types according to the stage of mineralization (Figure 1B): the first one is new, containing the newly built calices and inter-septal spaces at the surface layer, whereas the other is older, containing the older inter-septal spaces inside the colony. The newly formed calices and inter-septal spaces are at the surface of the coral and they are closely spaced, with small gaps indicating the existence of dissepiments (Wells, 1969). The basic morphological structure of the inner (older) inter-septal space is like those of the new ones, but their dissepiments are thinner. This phenomenon shows that, along with the colony growth via asexual reproduction, the dissepiments between adjacent inter-septal spaces become thinner, indicating the process of coral skeletal formation.

During the process of reconstruction of *P. damicornis*, we notice an important feature of the species that shows that the living polyps extend to the surface of the coral from their initial growth points (budding sites), whereas no polyp dies in the internal part of the coral. This indicates that coral growth is holistic at the macro level, although individual polyps are shown to be physically isolated from each other at the micro level. In addition, we identified several distinct growth types of *P. damicornis* according to the shape of calices and inter-septal spaces, including budding, nonbudding, and stunted growth types (Figure 2A). The budding and nonbudding types contain more inter-septal spaces than the stunted type. The budding type can undergo asexual reproduction, whereas the nonbudding type cannot. The inter-septal spaces of stunted growth type are much shorter than those two growth-type calices and are distributed near the surface of the colony. There are only a few calice and inter-septal space in the stunted type during the growth of the polyps.

To further investigate the growth pattern of coral polyps, we reconstructed the interior calices and inter-septal spaces of a smaller specimen in precise detail (Figure 2). We reconstructed a complete inter-septal space from its original growth point to the surface calice of the colony in a coral branchlet. We name a group of polyps with synchronized growth as “growth bundle” (Figure 2A). The growth bundle shown in Figure 2 contains all of the growth types described above. The budding growth-type polyps are most commonly found in the interior of the bundle, which is the point of origin of coral growth. The nonbudding growth-type polyps are at the periphery of the bundle. The newly budded polyps have relatively small, inverted cone-shaped inter-septal spaces, and they grow gradually into larger ones (Figure 2C). The inverted cone-shaped inter-septal spaces are important to discern the budding of polyps and are fundamental for the 3D reconstruction relationship of polyps in *P. damicornis* at a large scale, including the budding, growth, and distribution of polyps that form an entire interactional network, which we called internal calice and inter-septal space network. This characteristic makes it possible to capture the behaviors of all the polyps in one corallum of *P. damicornis* by reconstruction, and budding sites can be accurately recorded regardless of the fate of certain individual polyp.

The vertical growth of *P. damicornis* shows a clear direction. We noticed that each *P. damicornis* polyp grows along an axis. The direction of the axis points to the direction of the growth of the branch. Each polyp grows along the growth axis rather than toward the light directly. Polyps growing in the direction of the growth axis will continuously build new calices, whereas those polyps deviating from the growth axis commonly stop after four or five inter-septal spaces to mineralize the corallite; therefore, the side branches of *P. damicornis* become thickened. When the colony of *P. damicornis* branches, its growth axis is also divided, suggesting that the polyps in each of the new branches grow into different directions. We selected each polyp calice at the top of the branch and traced the growth process of each polyp. In the reconstruction model, we build the large-scale internal growth axis structure of *P. damicornis* and present the growth direction of each branch (Figure 2A). It is found that not all growth axes are sunlight-oriented. The polyps grow toward the direction that maximizes the light-receiving area of the entire colony to improve the sunlight utilization and survive under strong competitions.

**Figure 3:**
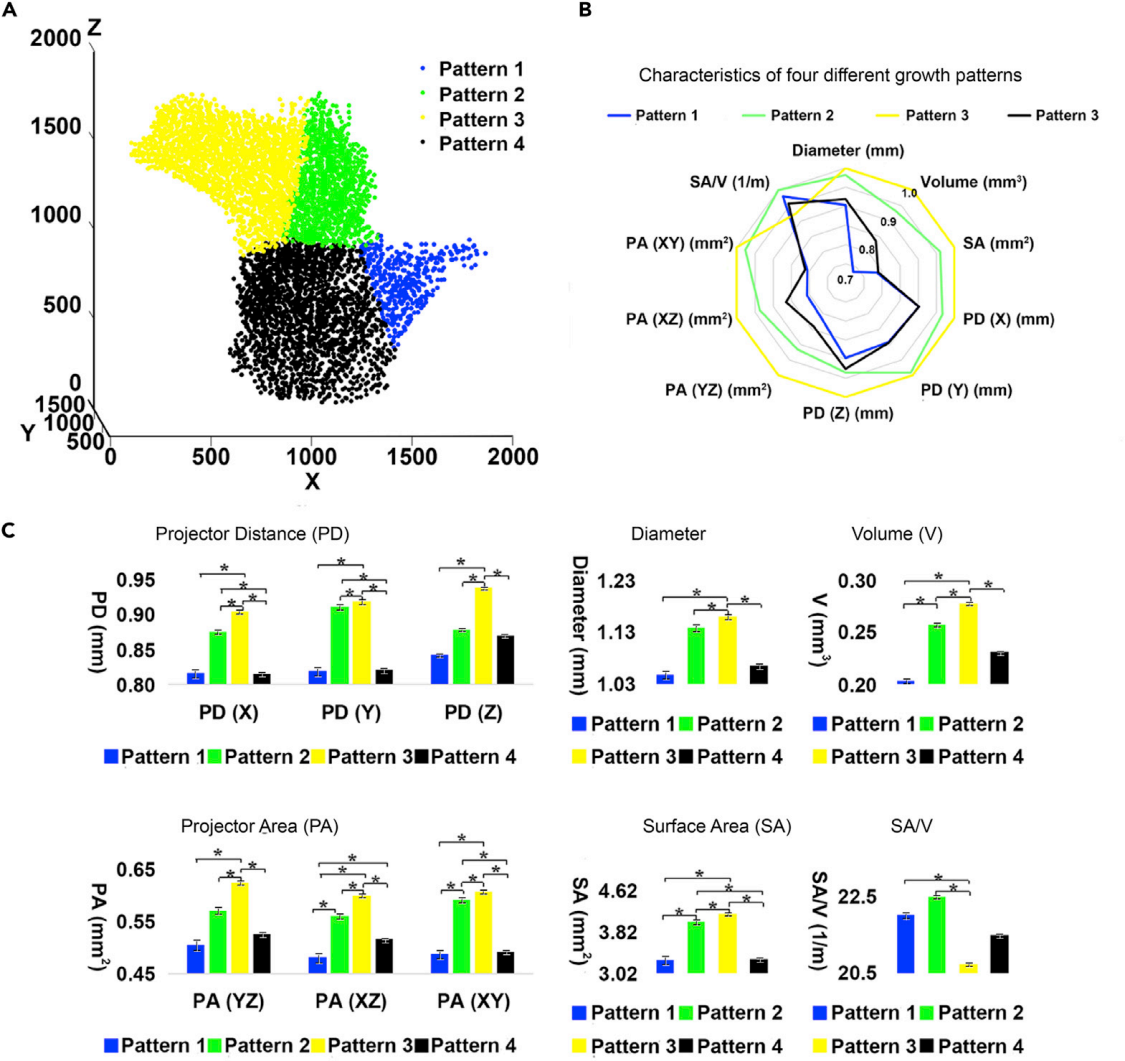
Cluster Analysis of *P. damicornis* Calice and Inter-Septal Space Obtained by Iterative Self-Organizing Data Analysis (ISODATA) (A) We analyze the reconstruction calices and inter-septal spaces of coral branchlets (shown in Figure 1B) from the perspectives of their diameter; volume; surface area; surface-volume ratio; spatial coordinates of the X, Y, and Z axes; projection distances along the X, Y, and Z axes; and projected areas on the XY, XZ, and YZ planes. The branchlet reconstruction models are divided into four sections: (1) low light mineralization area, (2) low light growth area, (3) light growth area, (4) light mineralization area. (B) Radar chart of the four sections. Growth areas 2 and 3 have higher average measurements than mineralization areas 1 and 4, indicating that mineralization accumulates and fills the inter-septal spaces of the coral skeleton. Light areas 3 and 4 have higher average measurements than low light areas 1 and 2, indicating that polyps exposed to light grow better and that light is significant for coral growth. (C) Ten histograms reflecting the data comparisons of four sections. We calculate the average of the data from 5,648 reconstruction calice and inter-septal spaces as the mean, whereas the error bars are standard deviation. The type of statistical test is independent-samples t test. “∗” marks in the figure means p < 0.05 between the two data groups.

We notice that all the coral branches split into two during corallite increase. There are no tripartite or any other kind of increase during the growth of the colony. Although many buds branch out along smaller growth axes, only two major growth directions remain under large-scale conditions (Figure 2A). Different environmental conditions will affect the internal calices and inter-septal spaces of branches on the same coral. Branchlet reconstruction models can be divided into four sections: (1) low light mineralization area, (2) low light growth area, (3) light growth area, and (4) light mineralization area (Figure 3 and Data S1). A comparison of measurement data from different sections shows that growth areas 2 and 3 have higher average values than those from the mineralization areas 1 and 4, indicating that mineralization accumulates and fills the inter-septal spaces of the coral skeleton. Light areas 3 and 4 have higher average volume, diameter, and surface area than those of the low light areas 1 and 2, indicating that polyps exposed to light build larger calices than those not exposed to light and show better growth activity.

A significant feature of the polyp behavior is synchrony in growth, in which all polyps spend a similar amount of time producing a chamber. The stratified structure of the skeleton is a direct result of this synchronous growth. To investigate this characteristic, we divided a branchlet into 48 layers and simulated the outline of coral polyps in each layer (Figure S4 and Video S1). We obtained coral growth rings by simulating the growth process from each of the 48 layers (Figure S4). We perform a statistical analysis of the diameter and volume of calices and inter-septal spaces between dissepiments in the 48-layer *P. damicornis* colony (Data S1), then we calculate the average diameters and volumes of the calices and inter-septal spaces in each layer and illustrate using line chats on these data (Figure S6; Table S1). Through this way, the growth ring-forming process of a reef-building coral has been visualized and is of great significance for the yearly, seasonal, and monthly growth studies of reef-building corals.

Video S1. Video Showing the Growth Process of a *P. damicornis* Branch, Related to Figure 2

## Discussion

### Growth Patterns of *P. damicornis*

The microscale structures of a corallum of *P. damicornis* is revealed by HRCT, which enables us to reconstruct a high-precision 3D model for this species (Figure 1). The rendered internal calices and inter-septal spaces of *P. damicornis* (Figures 1, 2, and S4) provide several insights on the formation of coral reefs. Our analysis of the skeleton and calice of *P. damicornis* shows that the corallite is sealed and calices are disconnected from each other. This finding demonstrates that the polyps grow independent from each other at the micro level.

However, although corallites are not related to each other at the micro level, they are uniformly regulated during growth because all living polyps extend to the surface of the coral from their initial growth point with no polyp death present in the internal part of the coral. The growth pattern of *Pocillopora* can be recognized as generally three-stage growth structure based on growth bundles along the dichotomous growth axes that are assembled by multiple growth types (Figure 2). As an important aspect of coral growth, we consider a group of corallites that derives from the same corallite as a growth bundle, which contains all the above-described growth types, and the growth direction of the bundle depends on the growth axis direction of its branch.

Light plays a vital role during the growth of the corals. Different environmental conditions affected calice and inter-septal space structures within the same colony and they can be classified into low light and lighted mineralization areas, and low light and lighted growth areas based on modeling. The measurements in the calice and inter-septal space reconstruction (including diameter; volume; surface area; surface-volume ratio data; projected distances of the X, Y, and Z axes; and projected areas of the XY, XZ, and YZ planes) show apparent differences between light areas and low-light areas (Figure 3). The data show that polyps in light areas grow better than those in dark areas, reflected by larger average volume and surface area of calices and inter-septal spaces. The direction of coral growth axis also goes toward the light area, whereas the growth of the dark area slows gradually and eventually stops. Because all these calices and inter-septal spaces are in the same branchlet, which means that their nutrient availability and water-current direction are basically the same, this analysis demonstrates the importance of lights to coral growth. However, this does not mean that all polyps grow straight toward the light; instead, all polyps in a colony grow along the growth axis of their branch. Once the growth deviates from the growth axis, the polyp stops growing, immediately secrets its new calice. *P. damicornis* also has a macroscopic integral growth axis that contains branches. Polyps in a new branch grow along the new axis. In this growth pattern, the active area is exposed to light increases, which significantly enhances the light energy utilization of the coral and plays a vital role in the competition of lights against other marine organisms of the same ecological niche. This phenomenon reveals the relationship between the coral formation process and the adaptive evolution of corals in enhancing photosynthesis.

During the process of coral growth, new buddings grow chamber by chamber. When a polyp secrets a new calice, the polyp enters a newly created chamber. A macroscopic result of the formation of new chambers is a growth ring (Figure S4). Because adjacent calices share skeletons, adjacent polyps in the same growth ring have to maintain a synchronized growth rate. In this study, we visualize the growth ring of *Pocillopora*. Addressing the growth of other reef-building corals is of great interest, if we are to understand their growth patterns at monthly, seasonal, and yearly time scales in detail.

### Calice-Reconstruction as a Method for Studying the Reef-Building Corals

Because the corallite structures and calice and inter-septal space network of *Pocillopora* are neat, we used *Pocillopora* as a model species to describe the method. The polyps in a *Pocillopora* colony build new calices from its budding point, which are significantly shown as inverted cone-shaped structures in the reconstruction. The process of polyp growth can be traced by studying the internal structure of the calices and inter-septal spaces; therefore, we can obtain the budding, growth, and distribution information of the polyps in a colony by reconstructions. Our study first records the growth patterns of *P. damicornis* based on the internal calice and inter-septal space network, which is poorly known from previous studies (Escalona et al., 1999, Bosch et al., 2010, Vizel et al., 2011). During the budding process of typical hydrozoans such as hydras and jellyfish (Schlesinger et al., 2010, Bosch, 2012), the new bud grows gradually upward, separated from the parents once it has a complete structure. In studies of the polyp network in these organisms, new buds are almost impossible to be traced after they are separated, making the growth and budding network of hydrozoans difficult to be studied. However, our study of *P. damicornis* by HRCT points out a potential solution to investigate the growth process of hydrozoans and their budding patterns.

Branches of *P. damicornis* are always dichotomous during the growth of the colony. There are no other exceptions observed in this study. The calice and inter-septal space of *P. damicornis* record the growth process of extending polyps; therefore, we can reconstruct the gradual growth of a colony. This investigation on the detailed growth process of *P. damicornis* by HRCT demonstrates the value of such method for understanding the coral body plan and the growth pattern of reef-building coral species.

### Conclusions

Our work presents an application of cutting-edge technology in investigating reef-building coral structures and reconstructing the internal calice and inter-septal space network, based on a large corallum of *P. damicornis*. We studied the growth patterns of *P. damicornis* through the budding, growth, and distribution information obtained from the reconstruction of coral calice and inter-septal space network. It reveals that each calice of *P. damicornis* is a basic polyp growth unit, and growth bundles are arranged along the growth axis in a dichotomous branching pattern. The growth pattern of *P. damicornis* lays the foundation for further explorations on the monthly, seasonal, and yearly growth of reef-building corals. Furthermore, we reconstruct the entire calice and inter-septal space network in *P. damicornis* by HRCT, which sheds light on studying the process of coral formation and budding patterns of living polyps. Our results also suggest that HRCT can be used to characterize the development and process of coral growth. To sum up, the three-dimensional morphological reconstruction on the calice and inter-septal space structures in *P. damicornis* reveal a method for studying the biological characteristics and growth patterns of reef-building coral species.

### Ethics

All coral sample collecting and processing were performed according to the local laws governing the welfare of invertebrate animals and were approved by the Southeast University (SEU) ethical committee.

### Limitations of the Study

It should be noted that the growth patterns listed in this study are based on the *P. damicornis* colony, so they may not be suitable for all coral species in *Pocillopora*. The method for studying the growth and budding patterns of coral species listed in this study can be used for many major reef-building coral genera like *Pocillopora*, *Acropora*, *Montipora*, and *Seriatopora.* However, there still could be some special coral species that cannot be studied through this method.

### Resource Availability

#### Lead Contact

Further information and requests for resources and reagents should be directed to and will be fulfilled by the Lead Contact, Zuhong Lu (zhlu@seu.edu.cn).

#### Materials Availability

The HRCT data in this study have been deposited to the IVPP Digital data repository ADMorph [Archives of Digital Morphology, https://doi.org/10.12112/F.15].

#### Data and Code Availability

The CT data that support the findings of this study, as well as the 3D surface files, are available in the IVPP Digital data repository ADMorph (Archives of Digital Morphology, https://doi.org/10.12112/F.15).

The code used in ISODATA test can be found in Supplemental Information.

## Methods

All methods can be found in the accompanying Transparent Methods supplemental file.

## Supplemental Information

Document S1. Transparent Methods, Figures S1–S6, and Table S1

Data S1 Excel File on the Statistical Analysis of Diameter and Volume of Calices between Dissepiments in the 48-layer *P. damicornis* Colony

## Footnotes

Supplemental Information can be found online at https://doi.org/10.1016/j.isci.2020.101069.
