# Supplementary material for: The 3D Reconstruction of Pocillopora Colony Sheds Light on the Growth Pattern of This Reef-Building Coral
Source: iScience. 2020 Apr 18;23(6):101069. doi: 10.1016/j.isci.2020.101069 (PMC7276440; doi:10.1016/j.isci.2020.101069)
Supplement: Document S1. Transparent Methods, Figures S1–S6, and Table S1 [file mmc1.pdf]

iScience, Volume *23*

Supplemental Information

The 3D Reconstruction of *Pocillopora* Colony Sheds Light on the Growth Pattern of This Reef-Building Coral

Yixin Li, Tingyu Han, Kun Bi, Kun Liang, Junyuan Chen, Jing Lu, Chunpeng He, and Zuhong Lu

1. Transparent Methods

Specimen Collection

P. damicornis in this study was collected from the South China Sea in 2018 (Figure S1). The coral sample was kept whole and housed in our laboratory coral tank, where all conditions simulated its habitat in the South China Sea. This *P. damicornis* is lavender-red in colour. Its main branches are about 3.0 cm thick, spreading irregularly upward. In the wild, the polyps emerge from the calices on the coral surface and sway with the current. When external stimulation occurs, such as physical impact or removal from seawater, the polyps immediately shrink into their calices, eventually returning to their original state after a period.

Coral Culture System

P. damicornis is cultured in a standard RedSea® tank (redsea575, Red Sea Aquatics Ltd). The temperature is kept at 25° C and the salinity (Red Sea Aquatics Ltd) is 1.025. The culture system is maintained by a Protein Skimmer (regal250s, Reef Octopus), a water chiller (tk1000, TECO Ltd), three coral lamps (AI®, Red Sea Aquatics Ltd), two wave devices (VorTech^{TM} MP40, EcoTech Marine Ltd), and calcium reactor (Calreact 200, Reef Octopus) etc.

X-ray Microtomography

We analysed three specimens (specimens 2 and 3 were separated from specimen 1) from the South China Sea. All CT scanning was performed using a v|tome|x M (General Electric, Milwaukee, WI) at Yinghua NDT, Shanghai, China.

Specimen 1 was scanned with a beam energy of 210 kV and a flux of 205 μA at a detector resolution of 87 μm per pixel, using a 360° rotation with a step size of 0.18°. A total of 2,000 transmission images were reconstructed in a 2,000 × 2,000 matrix of 2,000 slices in a two-dimensional reconstruction software developed by GE. Specimen 2 was scanned with a beam energy of 220 kV and a flux of 120 μA at a detector resolution of 18 μm per pixel, using a 360° rotation with a step size of 0.164°. A total of 2,200 transmission images were reconstructed in a 2,000 × 2,000 matrix of 2,000 slices in a two-dimensional reconstruction software developed by GE. Specimen 3 was scanned with a beam energy of 180 kV and a flux of 130 μA at a detector resolution of 16 μm per pixel, using a 360° rotation with a step size of 0.24°.

A total of 1,500 transmission images were reconstructed in a 2,000 × 2,000 matrix of 2,000 slices in a two-dimensional reconstruction software developed by GE. Image sections and 3D rendered images was performed using VG StudioMax Version 3.2 (Volume Graphics).

High-resolution computed tomography used in reef-building coral research

Our work presents a novel use of high-resolution computed tomography (HRCT) for studying reef-building coral structures. HRCT can nondestructively capture the appearance and internal structure of corals (Figure 1, Figure S5). Compared with traditional biological techniques, such as bailout, SEM, and grinding sections, HRCT has multiple advantages in this study.

First, this method does not require complicated and potentially destructive preparations such as pickling or fixing and can even be used directly on living corals. HRCT reveals the delicate internal skeletal structures in *P. damicornis* that are easily destroyed during electron microscope observation and grinding.

Second, traditional biological techniques are unable to determine the size of the polyp calice. Corrosion of the coral skeleton caused by pickling before grinding will distort measurement results, while the fixing process before electron microscopy also causes deformation of the coral skeleton. Polyps obtained by bailout will shrink upon the loss of the support of the skeleton and therefore do not display the true scale of the calice. However, we can effortlessly measure the internal coral skeleton width with microtomography, naturally obtaining the diameter of the polyp oral surface and the true size of the calice.

At the same time, high-resolution images can be obtained by microtomography, with resolutions up to 10 μm for samples of 10 cm^3 and resolutions of approximately 80 μm for large colonies of 3,600 cm^3, these resolutions are unmatched by other methods. Finally, because microtomography can capture all coral structural information in detail at once, we can observe any position and section in a colony as needed, saving coral resources and eliminating the burden of multiple measurements while deriving a complete analysis of a sample. Our results therefore suggest that large-scale microtomography technology can be used to characterize the development and progress of coral structural growth, filling in the gaps in current coral studies and making significant contributions to ecological biology research of marine organisms and habitats.

Scanning Electron Microscopy (SEM)

Polyp sample collection was run as previously described (Shapiro et al., 2016). A small branch tip (5-10 mm) is removed from the mother colony using a clean stainless-steel bone cutter. The branch tip is placed in an open glass Petri dish filled with filtered artificial seawater just covering the coral fragment. A gradual increasing salinity concentration results in a polyp bail-out response, which make polyps released from the coral skeleton due to water evaporation. One 5-10 mm *P. damicornis* branch can yield 30–40 polyps within 48 hours. The bail-out polyps were fixed with 2.5% glutaric dialdehyde in 0.035M PBSNa, and general SEM experimental steps were run as previously described (Chindapol et al., 2013).

Iterative Self-organizing Data Analysis (ISODATA)

ISODATA, which has been widely used as a clustering algorithm (Mingchao et al., 2017), is used to divide the calices and inter-septal spaces into different polyp growth patterns in this work. As an unsupervised classification algorithm, ISODATA has the advantage of permitting an unknown number of clusters (Boudraa et al., 1992; Velasco et al., 2007) to be specified rather than requiring that value to be known *a priori* in the k-means algorithm method (Ahmad et al., 2013). The ISODATA algorithm uses the following process: (1) set initial parameters. (2) Calculate the distance index function of each cluster. (3) Merge or split the clusters according to the given requirements. (4) Repeat iteratively, calculating new indexes and determining whether the results meet the clustering requirements (Code can be seen in the Supplemental Information). In this study, the input dataset is set as thirteen data layers for each coral chamber, including the diameter, volume, surface area, surface

area/volume, the spatial coordinates of the X, Y, and Z axes, the projected distance along the X, Y and Z axes, and projected areas on the XY, XZ and YZ planes. Then two sample t-tests are used for the characteristics of different patterns, including the diameter, volume, surface area, surface area/volume, projected distance along X, Y and Z axes and projected areas on the XY, XZ and YZ planes.

2. Supplemental Figures

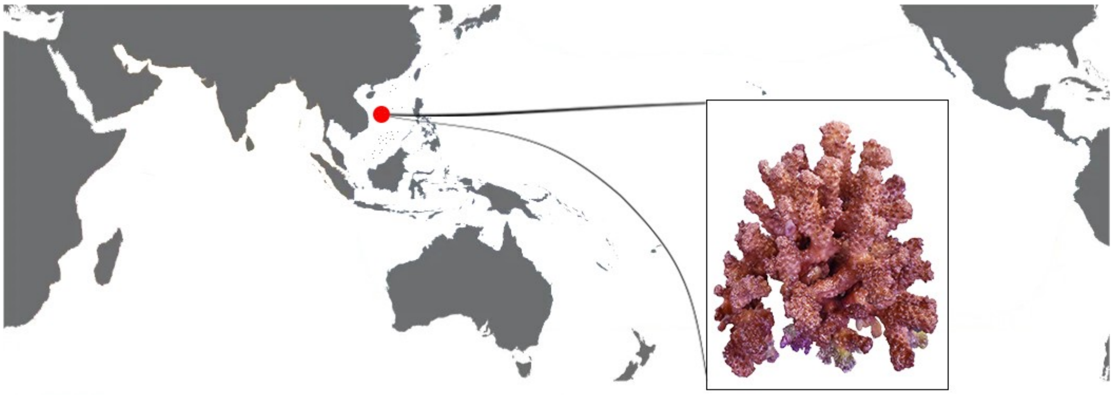

Figure S1 Sample location of the *Pocillopora* colony used in this study. Related to Figure 1 *P. damicornis* in this study was collected from the Xisha Islands (latitude 15°40'–17°10' north, longitude 111°—113° east), shown as a red dot at the centre of the map.

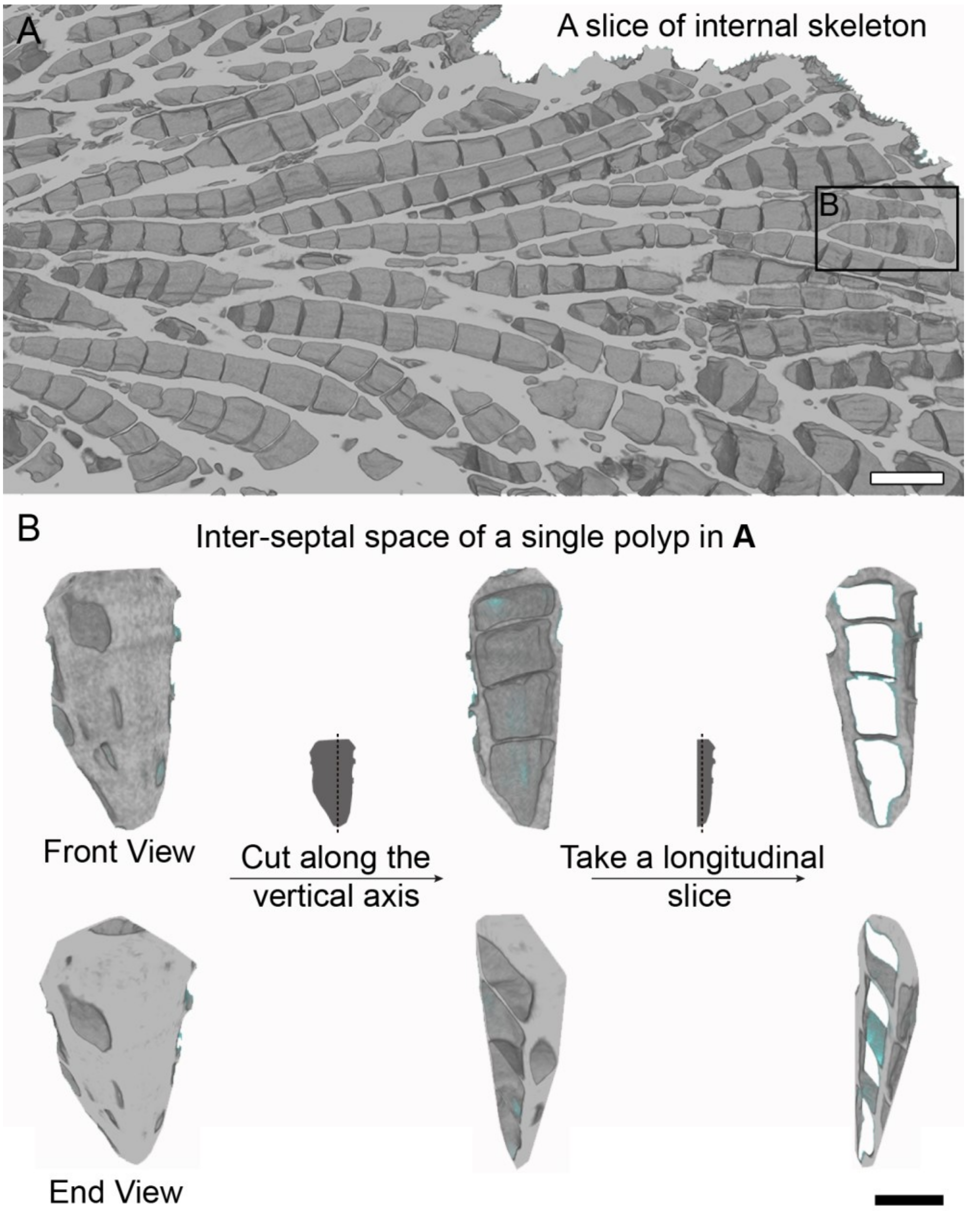

Figure S2 Schematic diagram of *P. damicornis* skeleton reconstruction. Related to Figure 1 (A) Sectional view of coral branch reconstruction. The bamboo-like calice and inter-septal space structures on the same branch is arranged in a generally alternate pattern. (B) We selected the calice and inter-septal space structures of a single polyp in **A**. To show its internal structure, we cut it along the vertical axis to expose the inter-septal spaces inside. For a more detailed observation, we took a longitudinal slice and find that there are some transparent sites along the extremely thin dissepiments, so that the various calices mineralized by one independent polyp are connected. Scale bars: (A) 1 mm; (B) 0.5 mm.

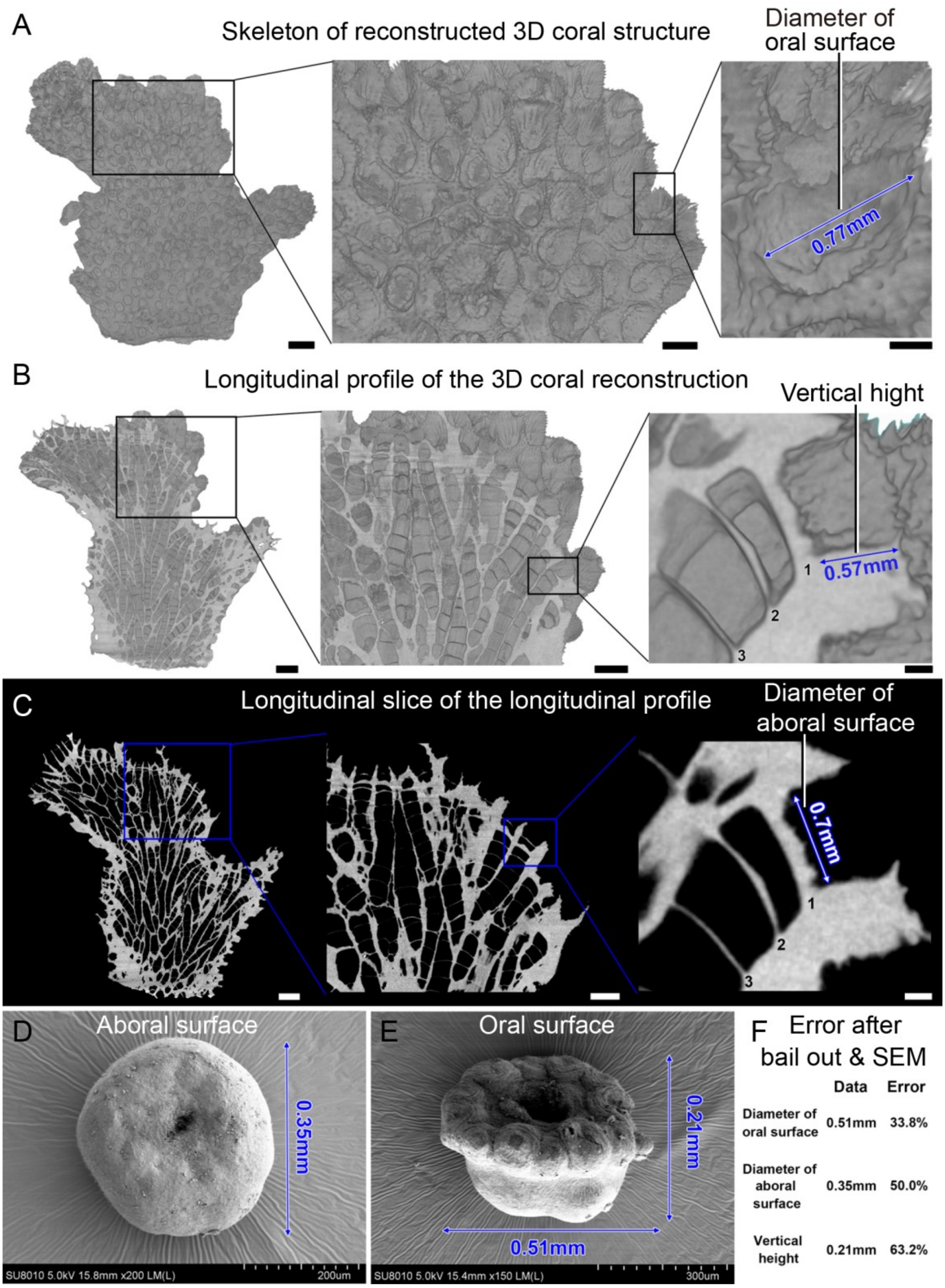

Figure S3 Related to Figure 1 Measuring the scale of a *P. damicornis* polyp calices with micro-CT. (A-C) We can directly measure the natural size of a calice in the 3D reconstruction, 3D profile and 3D slice produced by X-ray microfocus computed tomography. **A** is a reconstructed 3D structure, **B** is a longitudinal profile of the 3D reconstruction. **C** is a longitudinal slice of the longitudinal profile. (D, E) Scanning electron microscope (SEM) image of a *P. damicornis* polyp obtained by polypbail out (see Reference 1 for methodology). Polyps obtained by bailout shrink because they lose skeletal support, while the fixing process before SEM causes polyp

deformation, which distorts the true scale of the polyp calice. **D** is the SEM image of a polyp's abactinal surface. **E** is the SEM image of a polyp's oral surface. (F) Measuring error after polyp bailout and SEM. **A-C** Data are represented as mean ±HRCT. **D**, **E** Data are represented as mean ±SEM. Scale bars: (A) 2 mm, (B) 1 mm, (C) 0.2 mm; (D) 200 μm; (E) 300 μm.

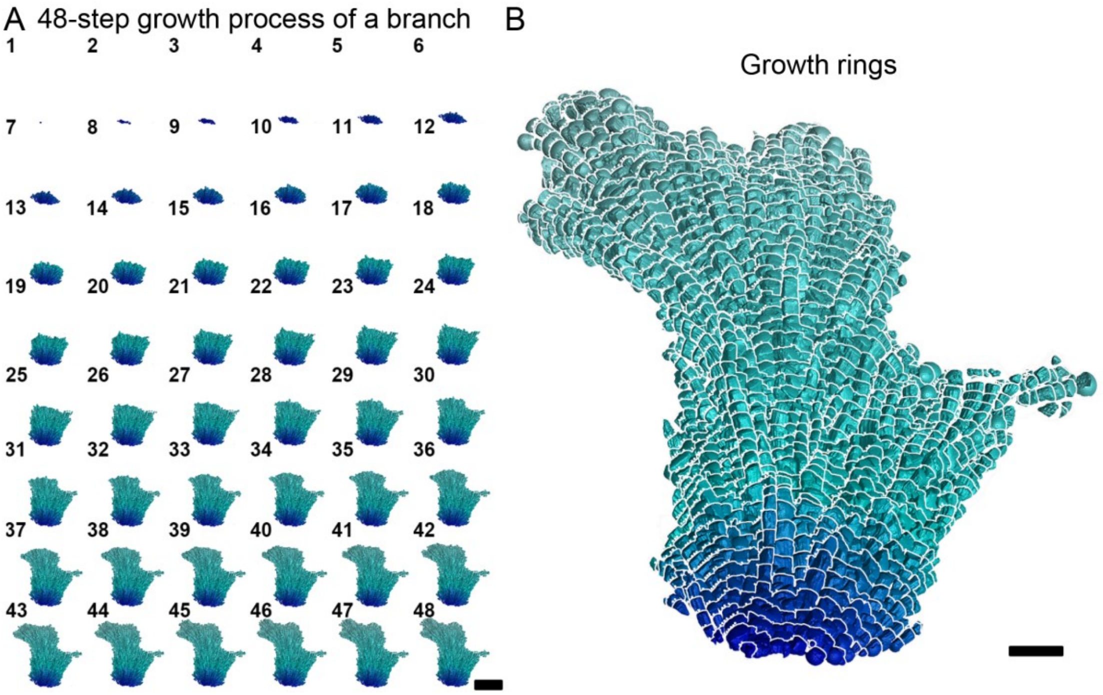

Figure S4 The 48-step growth process and growth rings of a branch of *P. damicornis*. Related to Figure 2 (A) By reconstructing all the pore canals in a single *P. damicornis* branch, and dividing the entire growth process into 48 strata according to the coral branch's horizontal synchronisation, we can present the growth process of *P. damicornis* directly. (B) By simulating the 48-step growth process of a *P. damicornis* branch, we can visualise its growth rings, showing how the *Pocillopora* grew and enabling research into the yearly, seasonal, and monthly growth of reef-building corals. Scale bars: (A) 1 mm; (B) 2 mm.

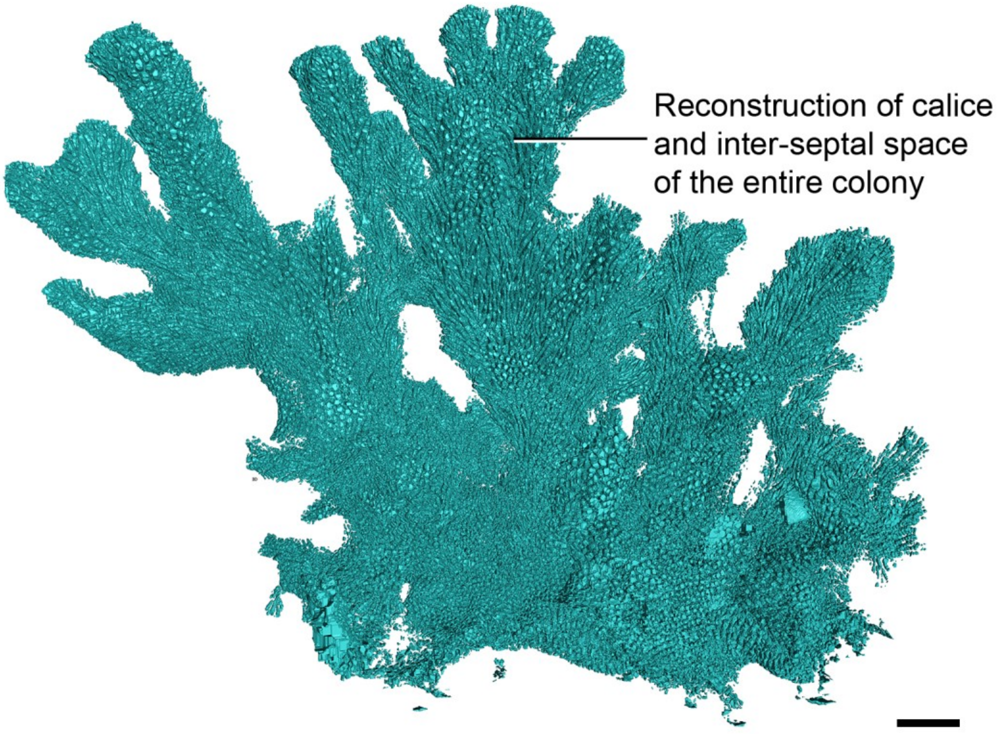

Figure S5 The reconstruction of calice and inter-septal space of the entire colony in Fig. 1A. Related to Figure 2 Scale bar: 1 cm.

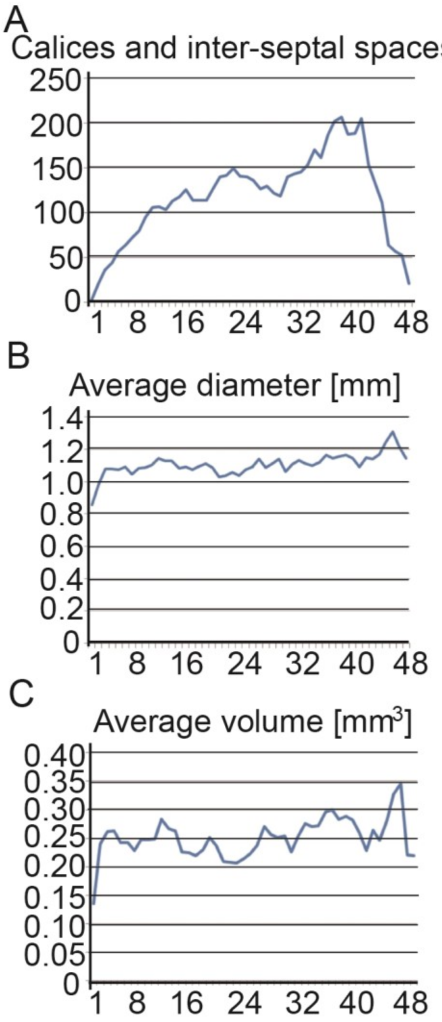

Figure S6 Line charts of calices, average diameter and average volume of calices in 48 layers. Related to Figure 3 (A) Calices and inter-septal space line chart. (B) Average diameter line chart. (C) Average volume line chart. Data are represented as mean ±HRCT.

3. Supplemental Table

Supplementary Table 1 Related to Figure 3 | Everage diameter and volume of calices between dissepiments in the 48-layer *P.damicornis* colony. Data are represented as mean ±HRCT.

Layer	Calices and inter-septal space	Average diameter [mm]	Average volume [mm^3]
1	2	0.855	0.135
2	21	0.986	0.240
3	36	1.077	0.262
4	44	1.079	0.263
5	57	1.073	0.243
6	64	1.092	0.243
7	72	1.045	0.229

8	80	1.082	0.248
9	95	1.088	0.248
10	106	1.103	0.249
11	107	1.146	0.283
12	104	1.126	0.267
13	113	1.127	0.263
14	118	1.081	0.226
15	126	1.090	0.225
16	114	1.073	0.219
17	114	1.095	0.230
18	114	1.110	0.252
19	127	1.086	0.237
20	140	1.031	0.210
21	142	1.038	0.208
22	150	1.058	0.207
23	141	1.038	0.214
24	140	1.075	0.223
25	136	1.089	0.237
26	127	1.141	0.271
27	130	1.087	0.257
28	122	1.110	0.251
29	119	1.141	0.254
30	140	1.064	0.226
31	143	1.108	0.253
32	146	1.133	0.276
33	153	1.113	0.271
34	170	1.101	0.272
35	162	1.118	0.296
36	187	1.166	0.300
37	202	1.143	0.284
38	207	1.157	0.288
39	188	1.166	0.282
40	189	1.143	0.261
41	205	1.092	0.228
42	154	1.148	0.264
43	134	1.140	0.247
44	112	1.171	0.281
45	64	1.247	0.327
46	57	1.309	0.346
47	53	1.214	0.221
48	21	1.146	0.220

4. Supplemental Code

Data S1 Related to Figure 3 | The entire code of ISODATA analysis:

```
function [centroid, result] = ISODATA(data, iteration, desired_k, minimum_n,
maximum_variance, minimum_d,centroid)

% Pre-allocate result
distance_matrix = zeros(size(data,1), desired_k);
result = zeros(size(data,1),1);

for i = 1 : iteration
    previous_centroid = centroid;
    for j = 1 : size(distance_matrix,1)
        for k = 1 : size(distance_matrix,2)
            distance_matrix(j,k) = sqrt(sum((data(j,:)-centroid(k,:)) .^ 2));
        end
    end
    [~,result] = min(distance_matrix,[],2);

    % Whether the number of points in each class is smaller than minimum_n
    for j = 1 : size(centroid, 1)
       if(isempty(find(result == j,1)) || (size(find(result == j),1) < minimum_n))

            % One class with number of points in it less than minimum_n
            % should be deleted, along with records in the distance_matrix
            centroid(j,:) = [];
            distance_matrix(:,j) = [];

            % Re-assign each point to its closet neighbor class
            [~,result] = min(distance_matrix,[],2);
```

```
            % Recalculate centroids
            for k = 1:size(centroid,1)
                centroid(k,:) = mean(data(result(:,1) == k,:));
            end
        end
    end

    % Check if combining and splitting are needed
    % Case 1: too few classes
    if(size(centroid,1) <= (desired_k/2))
        % Split
        [centroid] = ISODATA_split(data, centroid, result, minimum_n,
maximum_variance);

    % Case 2: too many classes
    elseif(size(centroid,1) > (2*desired_k))
        % Combine
        [centroid] = ISODATA_combine(centroid, result, minimum_d);

    end

    if(previous_centroid == centroid)
        fprintf('Clustering over after %i iterations...\n', i);
        break;
    end
end
```

```
end

% Splitting
function [centroid] = ISODATA_split(data, centroid, current_result, minimum_n,
maximum_variance)

[centroid_x, centroid_y] = size(centroid);

variance_matrix = zeros(centroid_x, centroid_y); % pre-allocate the variance matrix

for i = 1 : centroid_x
    for j = 1 : centroid_y
        variance_matrix(i,j) = var(data(current_result == i,j));
    end
end

class_variance = max(variance_matrix,[],2); % find the greatest one-dimension
variance per class

for i = 1 : centroid_x
    if((class_variance(i,1) > maximum_variance) && size(find(current_result ==
i),1) > (2*minimum_n))

        % The current class should be splitted into two different classes
        centroid(i,:) = centroid(i,:) + sqrt(maximum_variance);
        centroid(end+1,:) = centroid(i,:) - sqrt(maximum_variance); % add one new
class to centroid set
    end
```

```
end
end

% Combining
function [centroid] = ISODATA_combine(centroid, current_result, minimum_d)
centroid_x = size(centroid,1);
class_distance_matrix = zeros(centroid_x, centroid_x);

% Calculate distances between two different classes
for i = 1 : x
    for j = 1 : x
        if(i ~= j)
            class_distance_matrix(i,j) = sqrt(sum((centroid(i,:)-centroid(j,:)) .^ 2));
        end
    end
end

% Combining two classes
for i = 1 : x
    for j = 1 : x
        if((i ~= j) && (class_distance_matrix(i,j) <    minimum_d))
            n1 = size(find(current_result == i),1);
            n2 = size(find(current_result == j),1);
            centroid(i,:) = (1/(n1+n2)) * (n1 * centroid(i,:) + n2 * centroid(j,:));
            centroid(j,:) = [];
            break; % the number of combining operation is limited to 1 within
each iteration
```

end

end

end

End

6. Supplemental References

Shapiro, O. H., Kramarsky-Winter, E., Gavish, A. R., Stocker, R., Vardi, A. (2016). A coral-on-a-chip microfluidic platform enabling live-imaging microscopy of reef-building corals. *Nat. Commun*. **7**, 10860.

Chindapol, N., Kaandorp, J. A., Cronemberger, C., Mass, T., Genin, A. (2013). Modelling growth and form of the scleractinian coral *Pocillopora verrucosa* and the influence of hydrodynamics. *PLoS Comput. Biol.* **9**, e1002849.

Mingchao, L., Shuai, H., Jonathan S. (2017). An enhanced ISODATA algorithm for recognizing multiple electric appliances from the aggregated power consumption dataset. *Energy Build.* **140**, 305–316.

Boudraa, A. E. O. *et al*. (1992). Automatic left ventricular cavity detection using fuzzy ISODATA and connected-components labeling algorithms. *Conf. Proc. IEEE Eng. Med. Biol. Soc.* **2**, 1895–1896.

Velasco, F. R. D. (2007). Thresholding using the ISODATA clustering algorithm. *IEEE Trans. Syst. Man Cybern.* **10**, 771–774.

Ahmad, A., Sufahani, S. F. (2013). Analysis of Landsat 5^{TM} data of Malaysian land covers using ISODATA clustering technique. *Applied Electromagnetics*.
